# Supplementary material for: A metabolite sensor subunit of the Atg1/ULK complex regulates selective autophagy
Source: Nat Cell Biol. 2024 Feb 5;26(3):366–77. doi: 10.1038/s41556-024-01348-4 (PMC10940145; doi:10.1038/s41556-024-01348-4)
Supplement: Supplementary file 1 — Reporting Summary [file 41556_2024_1348_MOESM1_ESM.pdf]

nature portfolio

Corresponding author(s): Martin Graef

Last updated by author(s): Nov 1, 2023

Reporting Summary

Nature Portfolio wishes to improve the reproducibility of the work that we publish. This form provides structure for consistency and transparency in reporting. For further information on Nature Portfolio policies, see our Editorial Policies and the Editorial Policy Checklist.

Statistics

For all statistical analyses, confirm that the following items are present in the figure legend, table legend, main text, or Methods section.

n/a	Confirmed	
☐	☒	The exact sample size (*n*) for each experimental group/condition, given as a discrete number and unit of measurement
☐	☒	A statement on whether measurements were taken from distinct samples or whether the same sample was measured repeatedly
☐	☒	The statistical test(s) used AND whether they are one- or two-sided<br>*Only common tests should be described solely by name; describe more complex techniques in the Methods section.*
☒	☐	A description of all covariates tested
☐	☒	A description of any assumptions or corrections, such as tests of normality and adjustment for multiple comparisons
☐	☒	A full description of the statistical parameters including central tendency (e.g. means) or other basic estimates (e.g. regression coefficient) AND variation (e.g. standard deviation) or associated estimates of uncertainty (e.g. confidence intervals)
☐	☒	For null hypothesis testing, the test statistic (e.g. *F*, *t*, *r*) with confidence intervals, effect sizes, degrees of freedom and *P* value noted<br>*Give P values as exact values whenever suitable.*
☒	☐	For Bayesian analysis, information on the choice of priors and Markov chain Monte Carlo settings
☒	☐	For hierarchical and complex designs, identification of the appropriate level for tests and full reporting of outcomes
☒	☐	Estimates of effect sizes (e.g. Cohen's *d*, Pearson's *r*), indicating how they were calculated

Our web collection on statistics for biologists contains articles on many of the points above.

Software and code

Policy information about availability of computer code

Data collection	*Provide a description of all commercial, open source and custom code used to collect the data in this study, specifying the version used OR*
Data analysis	Proteomics raw data was analzyed using MaxQuant (1.5.3.17, 1.6.10.43, 2..0.1.0, or 2.3.0) using the integrated Andromeda search engine. Imaging data was processed using Fusion software (Andor) and Fiji ImageJ Version 2.1.0. Deconvolution was performed using Huygnes Professional 16.10. Western blot data was analyzed using ImageStudioLite Version 5.2.5. Sequence alignments were performed using Clustal Omega (1.2.4)

For manuscripts utilizing custom algorithms or software that are central to the research but not yet described in published literature, software must be made available to editors and reviewers. We strongly encourage code deposition in a community repository (e.g. GitHub). See the Nature Portfolio guidelines for submitting code & software for further information.

Data

Policy information about availability of data

All manuscripts must include a data availability statement. This statement should provide the following information, where applicable:

- Accession codes, unique identifiers, or web links for publicly available datasets
- A description of any restrictions on data availability
- For clinical datasets or third party data, please ensure that the statement adheres to our policy

The proteomics datasets shown in Fig. 1c (10.6084/m9.figshare.21751184), Fig. 2a (10.6084/m9.figshare.21751187), Fig. 2b (10.6084/m9.figshare.24450133), Fig.3 g-i (PXD045932), Fig. 4f and 4i (10.6084/m9.figshare. 21751190), Fig. 5c (10.6084/m9.figshare. 21751181) are available in the figshare repository.

Mass spectrometry data have been deposited to the ProteomeXchange Consortium via the PRIDE partner repository (PubMed ID: 34723319) with the dataset identifier PXD045932 and PXD048177. Databases used included Uniprot full-length yeast proteome.

Human research participants

Policy information about studies involving human research participants and Sex and Gender in Research.

Reporting on sex and gender	*not applicable*
Population characteristics	*not applicable*
Recruitment	*not applicable*
Ethics oversight	*not applicable*

Note that full information on the approval of the study protocol must also be provided in the manuscript.

Field-specific reporting

Please select the one below that is the best fit for your research. If you are not sure, read the appropriate sections before making your selection.

☒ Life sciences ☐ Behavioural & social sciences ☐ Ecological, evolutionary & environmental sciences

For a reference copy of the document with all sections, see nature.com/documents/nr-reporting-summary-flat.pdf

Life sciences study design

All studies must disclose on these points even when the disclosure is negative.

Sample size	A minimum of four (4) independent biological replicates were generated, except for the whole cell proteomics in Fig. 2a and the Co-IP MS in Fig 4f (n=3-4). A sufficient sample size was determined based on variance between experimental groups.
Data exclusions	Data were excluded from analysis only upon clear technical failure.
Replication	All experiments were replicated at a minimum of 4 independent biological replicates as indicated in figure legends.
Randomization	Proteomics samples were analyzed in randomized order (Fig. 1c, 2a, 4f, and 5c)<br>Analysis of imaging data was performed by randomly choosing cells from each replicate (Fig. 1e,f, 2f,g, 5e, Extended data 1a,c,d, e)<br>All other samples were analyzed based on genotypes, treatments, and/or time points with internal controls with randomization.<br>During fluorescence imaging, groups were imaged in random order to exclude positional effects in the imaging pipeline
Blinding	Mass spectrometry measurements and data analysis was performed and analyzed in a blinded manner. Fluorescence imaging was performed and analyzed in non-blinded manner, but independently verified by different experimentators.

Reporting for specific materials, systems and methods

We require information from authors about some types of materials, experimental systems and methods used in many studies. Here, indicate whether each material, system or method listed is relevant to your study. If you are not sure if a list item applies to your research, read the appropriate section before selecting a response.

Materials & experimental systems

n/a	Involved in the study
☐	☒ Antibodies
☒	☐ Eukaryotic cell lines
☒	☐ Palaeontology and archaeology
☒	☐ Animals and other organisms
☒	☐ Clinical data
☒	☐ Dual use research of concern

Methods

n/a	Involved in the study
☒	☐ ChIP-seq
☒	☐ Flow cytometry
☒	☐ MRI-based neuroimaging

Antibodies

Antibodies used

Primary antibodies: α-GFP (monoclonal, Takara, 632380), α-mCherry (polyclonal, Genetex, GTX128508), α-Atg13 (polyclonal, a gift from Prof. Daniel Klionsky) antibodies.
Secondary antibodies: α-mouse or α-rabbit Dylight800/680 antibodies (Rockland Immunochemicals, 610-745-124 and 611-142-122 respectively). **a–GFP (JL–8, monoclonal, Takara, 632380)**

Validation

α-GFP (monoclonal, Takara, 632380) https://www.takarabio.com/documents/Certificate%20of%20Analysis/632380/632380-632381-070313.pdf
α-mCherry (polyclonal, Genetex, GTX128508) https://www.genetex.com/PDF/Download?catno=GTX128508
α-Atg13 doi: 10.1074/jbc.M002813200 and doi: 10.4161/auto.27707
