# Supplementary figures and images for: A metabolite sensor subunit of the Atg1/ULK complex regulates selective autophagy
Source: Nat Cell Biol. 2024 Feb 5;26(3):366–77. doi: 10.1038/s41556-024-01348-4 (PMC10940145; doi:10.1038/s41556-024-01348-4)

**Figure 1b**

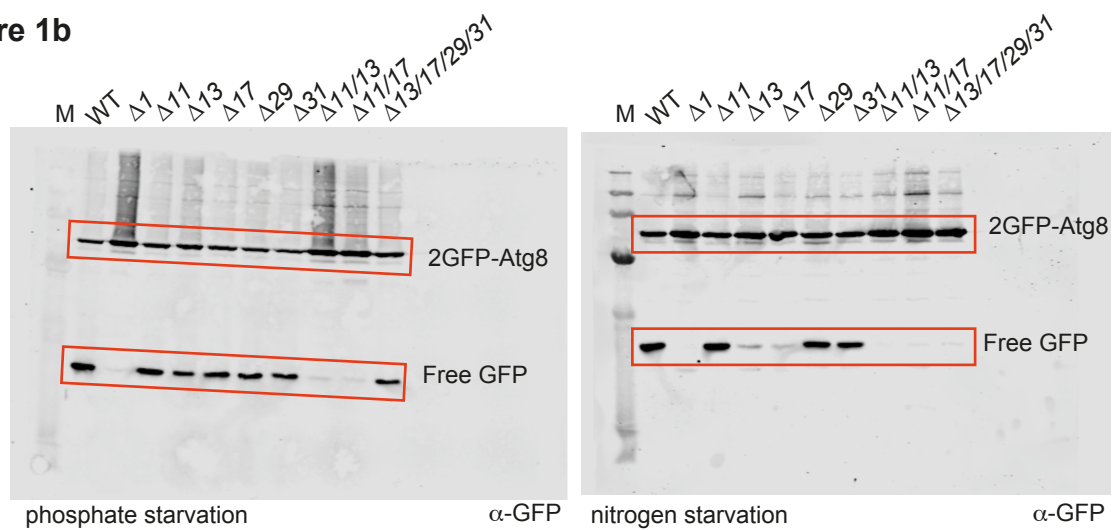

**Figure 1d**

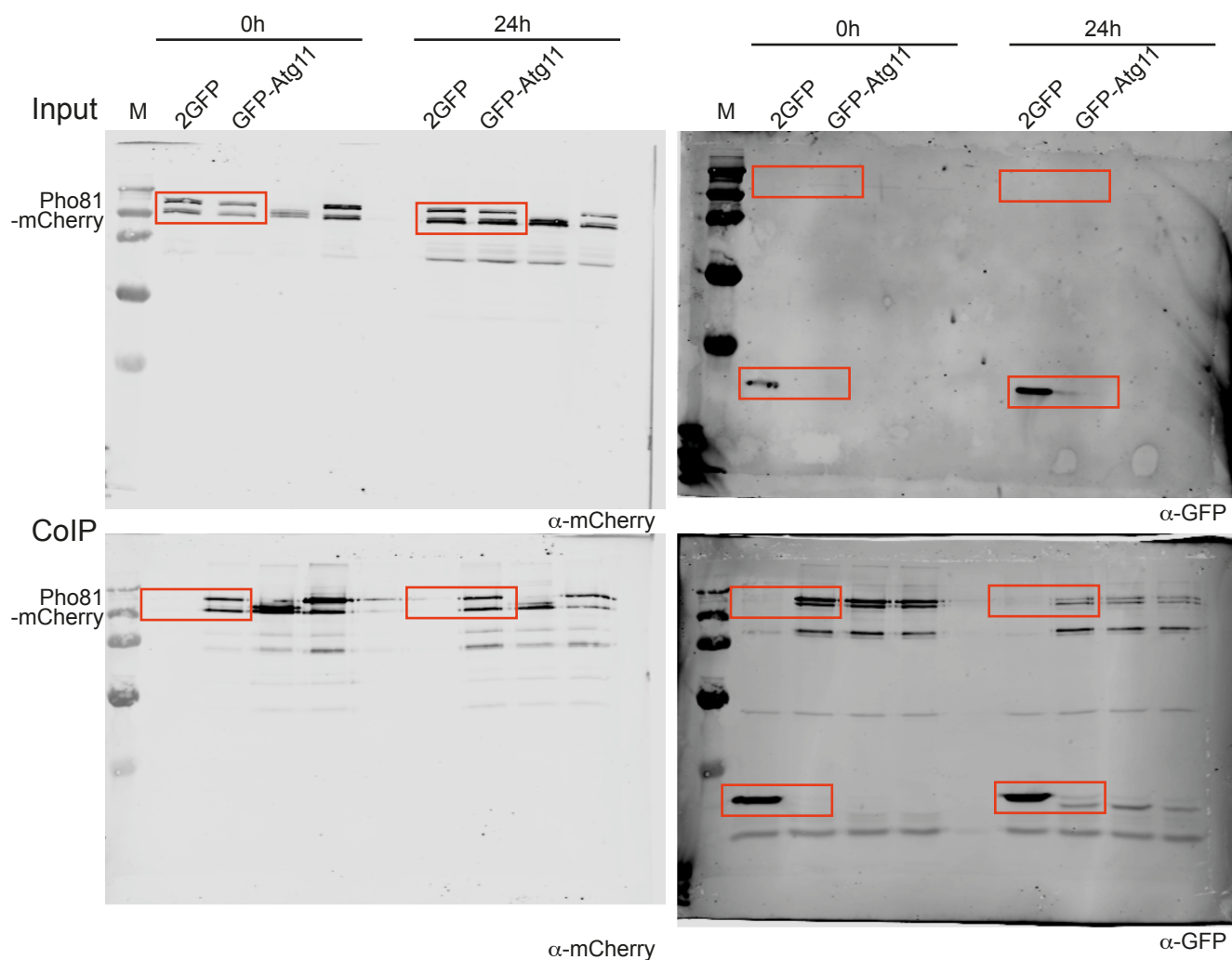

Supplement: Supplementary file 4 — Unprocessed western blots. [file 41556_2024_1348_MOESM4_ESM.pdf]

**Figure 2c**

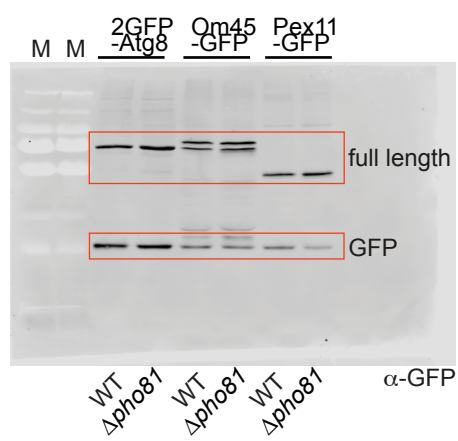

**Figure 2d**

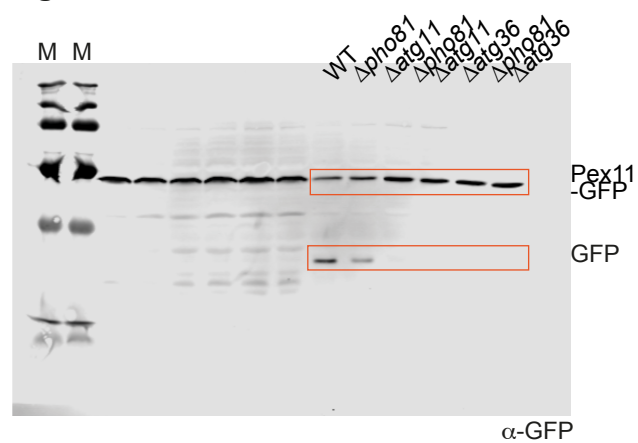

**Figure 2e**

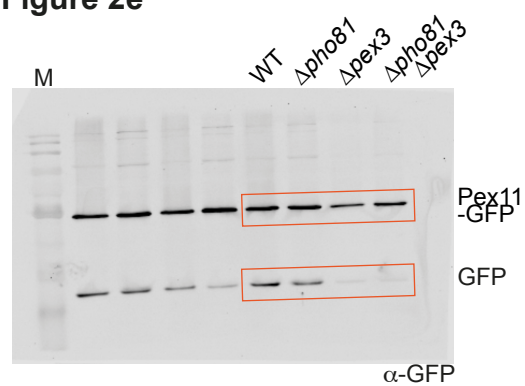

Supplement: Supplementary file 6 — Unprocessed western blots. [file 41556_2024_1348_MOESM6_ESM.pdf]

**Figure 3b**

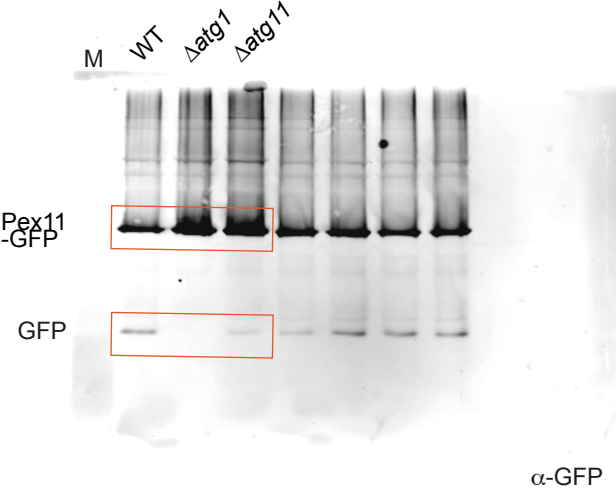

**Figure 3c**

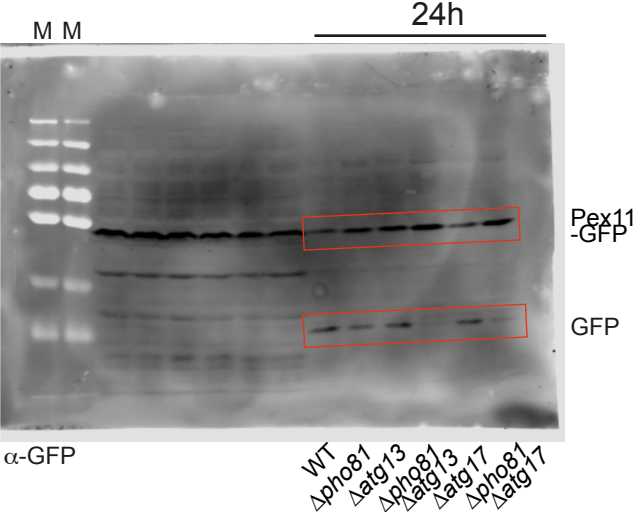

**Figure 3d**

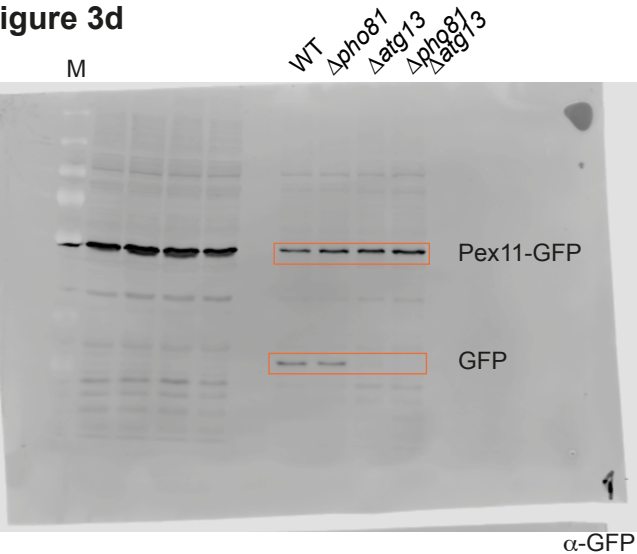

**Figure 3e**

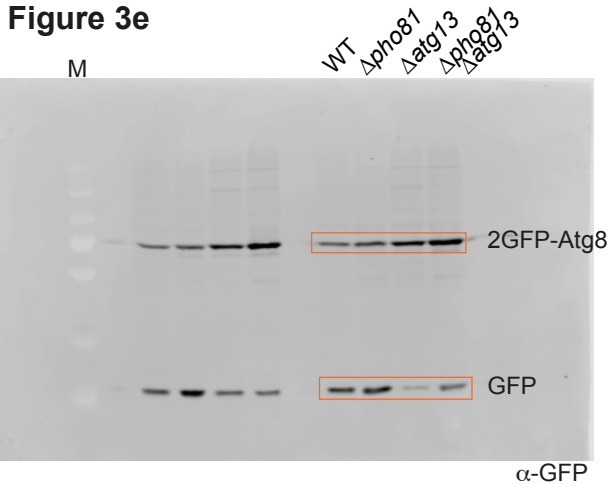

**Figure 3f**

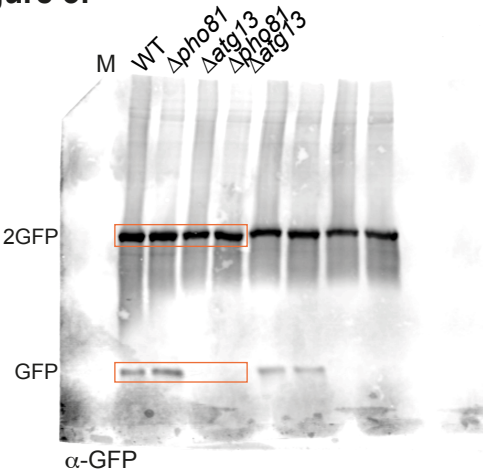

Supplement: Supplementary file 8 — Unprocessed western blots. [file 41556_2024_1348_MOESM8_ESM.pdf]

Figure 4d

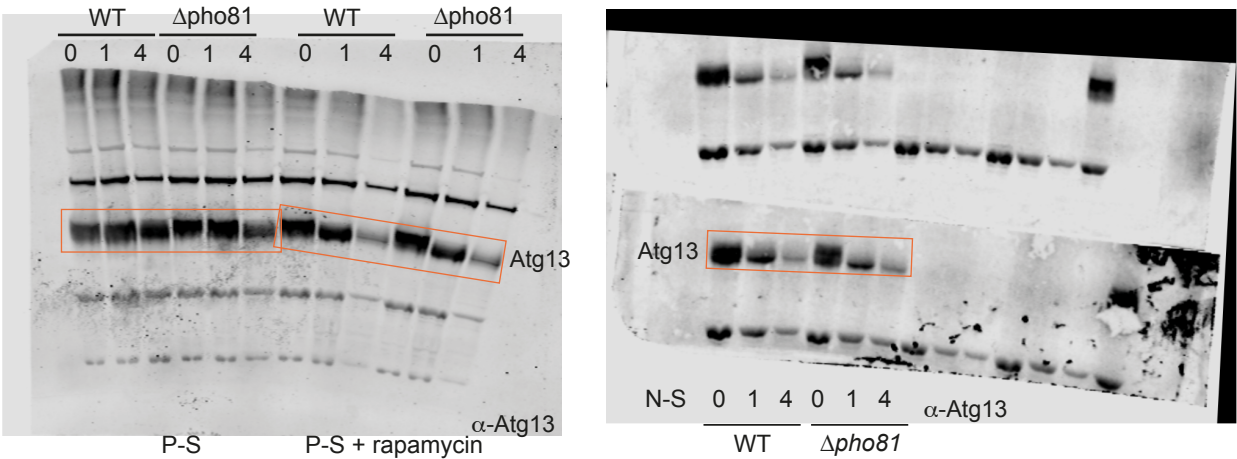

Figure 4e

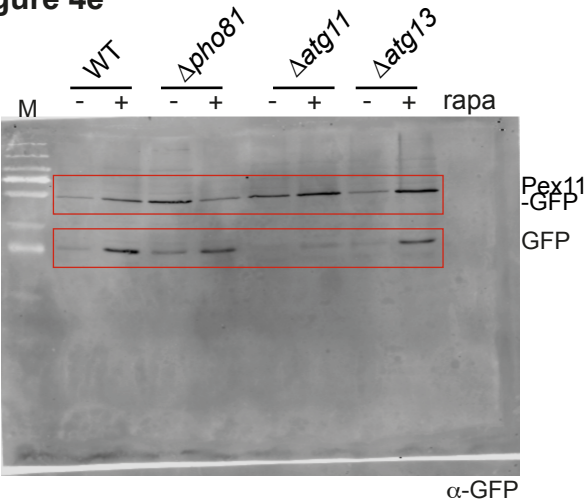

Figure 4f

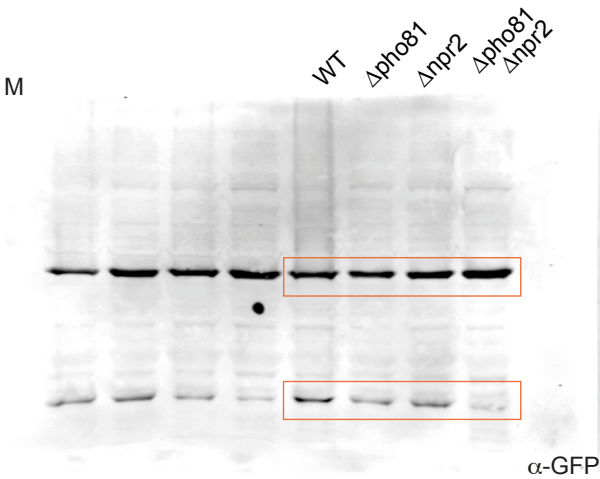

Supplement: Supplementary file 10 — Unprocessed western blots. [file 41556_2024_1348_MOESM10_ESM.pdf]

Figure 5g

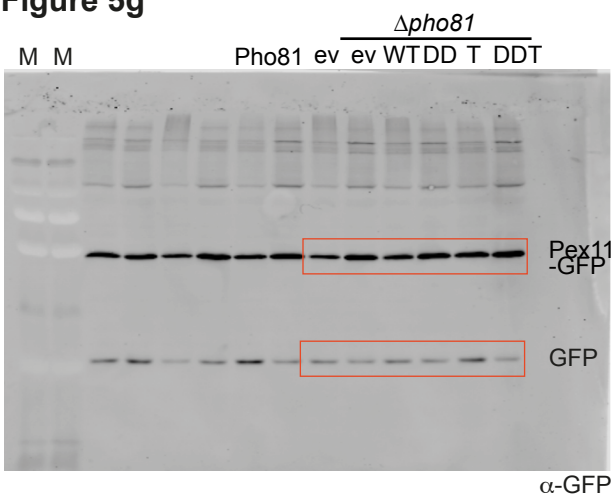

Figure 5h

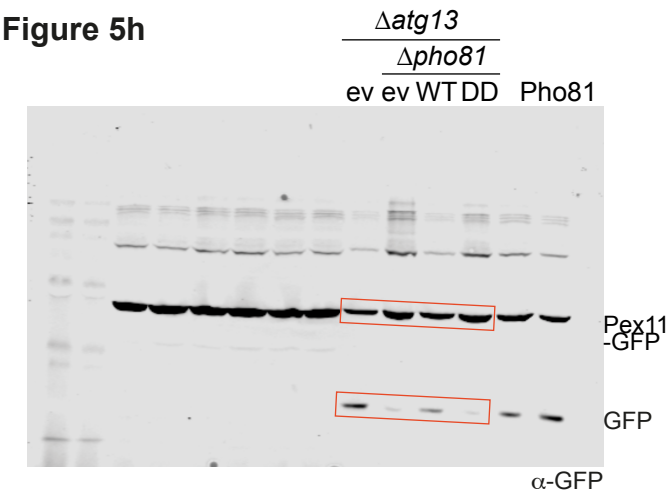

Figure 5j

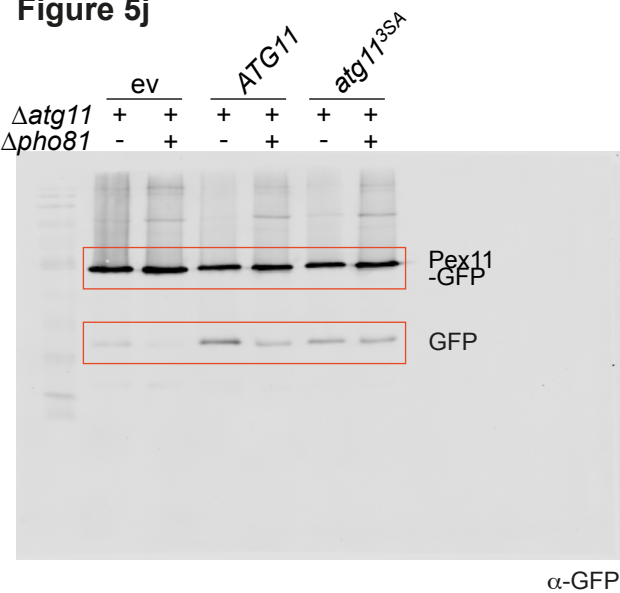

Supplement: Supplementary file 12 — Unprocessed western blots. [file 41556_2024_1348_MOESM12_ESM.pdf]

Figure 6d

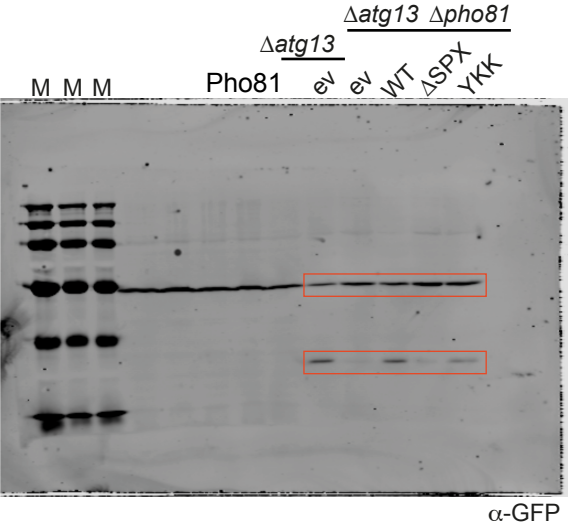

Figure 6g

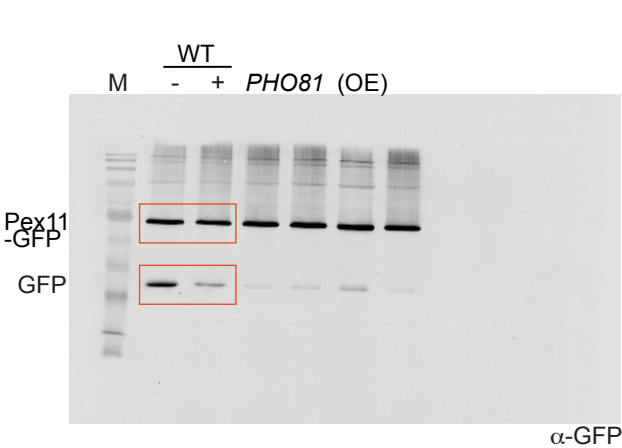

Figure 6h

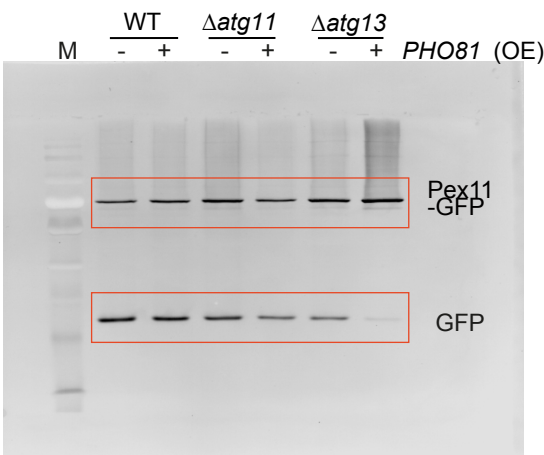

Supplement: Supplementary file 14 — Unprocessed western blots. [file 41556_2024_1348_MOESM14_ESM.pdf]

## Extended data 1f

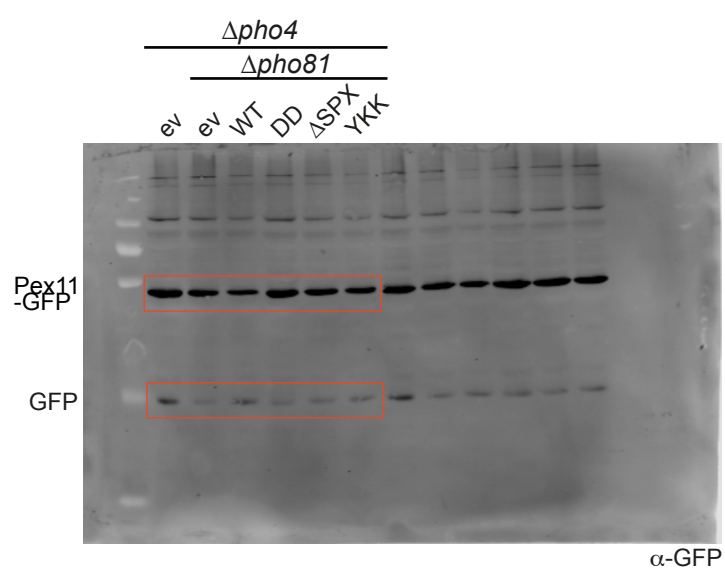

Supplement: Supplementary file 16 — Unprocessed western blots. [file 41556_2024_1348_MOESM16_ESM.pdf]
